# Large-scale genome-wide meta-analysis of polycystic ovary syndrome suggests shared genetic architecture for different diagnosis criteria

**DOI:** 10.1371/journal.pgen.1007813

**Published:** 2018-12-19

**Authors:** Felix Day, Tugce Karaderi, Michelle R. Jones, Cindy Meun, Chunyan He, Alex Drong, Peter Kraft, Nan Lin, Hongyan Huang, Linda Broer, Reedik Magi, Richa Saxena, Triin Laisk, Margrit Urbanek, M. Geoffrey Hayes, Gudmar Thorleifsson, Juan Fernandez-Tajes, Anubha Mahajan, Benjamin H. Mullin, Bronwyn G. A. Stuckey, Timothy D. Spector, Scott G. Wilson, Mark O. Goodarzi, Lea Davis, Barbara Obermayer-Pietsch, André G. Uitterlinden, Verneri Anttila, Benjamin M. Neale, Marjo-Riitta Jarvelin, Bart Fauser, Irina Kowalska, Jenny A. Visser, Marianne Andersen, Ken Ong, Elisabet Stener-Victorin, David Ehrmann, Richard S. Legro, Andres Salumets, Mark I. McCarthy, Laure Morin-Papunen, Unnur Thorsteinsdottir, Kari Stefansson, the 23andMe Research Team, Unnur Styrkarsdottir, John R. B. Perry, Andrea Dunaif, Joop Laven, Steve Franks, Cecilia M. Lindgren, Corrine K. Welt

**Affiliations:** 1 MRC Epidemiology Unit, Cambridge Biomedical Campus, University of Cambridge School of Clinical Medicine, Cambridge, United Kingdom; 2 The Wellcome Trust Centre for Human Genetics, University of Oxford, Oxford, United Kingdom; 3 Department of Biological Sciences, Faculty of Arts and Sciences, Eastern Mediterranean University, Famagusta, Cyprus; 4 Center for Bioinformatics & Functional Genomics, Department of Biomedical Sciences, Cedars-Sinai Medical Center, Los Angeles, California, United States of America; 5 Division of Reproductive Endocrinology and Infertility, Department of Obstetrics and Gynaecology, Erasmus MC, University Medical Center Rotterdam, Rotterdam, The Netherlands; 6 Department of Internal Medicine, University of Kentucky College of Medicine, Lexington, Kentucky, United States of America; 7 University of Kentucky Markey Cancer Center, Lexington, Kentucky, United States of America; 8 Departments of Epidemiology and Biostatistics, Harvard T.H. Chan School of Public Health, Boston, Massachusetts, United States of America; 9 Department of Internal Medicine, Erasmus MC, University Medical Center Rotterdam, Rotterdam, The Netherlands; 10 Estonian Genome Center, Institute of Genomics, University of Tartu, Tartu, Estonia; 11 Broad Institute of Harvard and MIT and Massachusetts General Hospital, Harvard Medical School, Boston, Massachusetts, United States of America; 12 Department of Obstetrics and Gynaecology, Institute of Clinical Medicine, University of Tartu, Tartu, Estonia; 13 Division of Endocrinology, Metabolism, and Molecular Medicine, Department of Medicine, Northwestern University Feinberg School of Medicine, Chicago, Illinois, United States of America; 14 Center for Genetic Medicine, Northwestern University Feinberg School of Medicine, Chicago, Illinois, United States of America; 15 Department of Anthropology, Northwestern University, Evanston, Illinois, United States of America; 16 deCODE genetics/Amgen, Reykjavik, Iceland; 17 Oxford Centre for Diabetes, Endocrinology and Metabolism, University of Oxford, Oxford, United Kingdom; 18 Department of Endocrinology & Diabetes, Sir Charles Gairdner Hospital, Nedlands, Western Australia, Australia; 19 School of Medicine and Pharmacology, University of Western Australia, Crawley, Western Australia, Australia; 20 Keogh Institute for Medical Research, Nedlands, Western Australia, Australia; 21 Department of Twin Research & Genetic Epidemiology, King's College London, London, United Kingdom; 22 Division of Endocrinology, Diabetes and Metabolism, Department of Medicine, Cedars-Sinai Medical Center, Los Angeles, California, United States of America; 23 Department of Medicine, Division of Genetic Medicine, Vanderbilt University Medical Center, Nashville, Tennessee, United States of America; 24 Vanderbilt Genomics Institute, Vanderbilt University Medical Center, Nashville, Tennessee, United States of America; 25 Division of Endocrinology and Diabetology, Department of Internal Medicine Medical University of Graz, Graz, Austria; 26 Stanley Center for Psychiatric Genetics, Broad Institute of MIT and Harvard, Cambridge, Massachusetts, United States of America; 27 Analytic and Translational Genetics Unit, Massachusetts General Hospital and Harvard Medical School, Boston, Massachusetts, United States of America; 28 Department of Epidemiology and Biostatistics, MRC-PHE Centre for Environment and Health, School of Public Health, Imperial College London, London, United Kingdom; 29 Center for Life Course Health Research, Faculty of Medicine, University of Oulu, Oulu, Finland; 30 Biocenter Oulu, University of Oulu, Oulu, Finland; 31 Unit of Primary Care, Oulu University Hospital, Oulu, Finland; 32 Department of Reproductive Medicine and Gynaecology, University Medical Center, Utrecht, The Netherlands; 33 Department of Internal Medicine and Metabolic Diseases, Medical University of Białystok, Białystok, Poland; 34 Department of Internal Medicine, Section of Endocrinology, Erasmus MC, University Medical Center Rotterdam, Rotterdam, The Netherlands; 35 Odense University Hospital, University of Southern Denmark, Odense, Denmark; 36 Department of Physiology and Pharmacology, Karolinska Institutet, Stockholm, Sweden; 37 Department of Medicine, Section of Adult and Paediatric Endocrinology, Diabetes, and Metabolism, The University of Chicago, Chicago, Illinois, United States of America; 38 Department of Obstetrics and Gynecology and Public Health Sciences, Penn State University College of Medicine, Hershey, Pennsylvania, United States of America; 39 Competence Centre on Health Technologies, Tartu, Estonia; 40 Institute of Bio- and Translational Medicine, University of Tartu, Tartu, Estonia; 41 Department of Obstetrics and Gynecology, University of Helsinki and Helsinki University Hospital, Helsinki, Finland; 42 Oxford NIHR Biomedical Research Centre, Churchill Hospital, Oxford, United Kingdom; 43 Department of Obstetrics and Gynecology, University of Oulu and Oulu University Hospital, Medical Research Center, PEDEGO Research Unit, Oulu, Finland; 44 Faculty of Medicine, University of Iceland, Reykjavik, Iceland; 45 Division of Endocrinology, Diabetes and Bone Disease, Icahn School of Medicine at Mount Sinai, New York, New York, United States of America; 46 Institute of Reproductive & Developmental Biology, Department of Surgery & Cancer, Imperial College London, London, United Kingdom; 47 Big Data Institute, Li Ka Shing Centre for Health Information and Discovery, Nuffield Department of Medicine, University of Oxford, Oxford, United Kingdom; 48 Division of Endocrinology, Metabolism and Diabetes, University of Utah, Salt Lake City, Utah, United States of America; 49 Reproductive Endocrine Unit, Massachusetts General Hospital, Boston, Massachusetts, United States of America; Yale School of Medicine, UNITED STATES

## Abstract

Polycystic ovary syndrome (PCOS) is a disorder characterized by hyperandrogenism, ovulatory dysfunction and polycystic ovarian morphology. Affected women frequently have metabolic disturbances including insulin resistance and dysregulation of glucose homeostasis. PCOS is diagnosed with two different sets of diagnostic criteria, resulting in a phenotypic spectrum of PCOS cases. The genetic similarities between cases diagnosed based on the two criteria have been largely unknown. Previous studies in Chinese and European subjects have identified 16 loci associated with risk of PCOS. We report a fixed-effect, inverse-weighted-variance meta-analysis from 10,074 PCOS cases and 103,164 controls of European ancestry and characterisation of PCOS related traits. We identified 3 novel loci (near *PLGRKT*, *ZBTB16 and MAPRE1*), and provide replication of 11 previously reported loci. Only one locus differed significantly in its association by diagnostic criteria; otherwise the genetic architecture was similar between PCOS diagnosed by self-report and PCOS diagnosed by NIH or non-NIH Rotterdam criteria across common variants at 13 loci. Identified variants were associated with hyperandrogenism, gonadotropin regulation and testosterone levels in affected women. Linkage disequilibrium score regression analysis revealed genetic correlations with obesity, fasting insulin, type 2 diabetes, lipid levels and coronary artery disease, indicating shared genetic architecture between metabolic traits and PCOS. Mendelian randomization analyses suggested variants associated with body mass index, fasting insulin, menopause timing, depression and male-pattern balding play a causal role in PCOS. The data thus demonstrate 3 novel loci associated with PCOS and similar genetic architecture for all diagnostic criteria. The data also provide the first genetic evidence for a male phenotype for PCOS and a causal link to depression, a previously hypothesized comorbid disease. Thus, the genetics provide a comprehensive view of PCOS that encompasses multiple diagnostic criteria, gender, reproductive potential and mental health.

## Author summary

We performed an international meta-analysis of genome-wide association studies combining over 10,000,000 genetic markers in more than 10,000 European women with polycystic ovary syndrome (PCOS) and 100,000 controls. We found three new risk variants associated with PCOS. Our data demonstrate that the genetic architecture does not differ based on the diagnostic criteria used for PCOS. We also demonstrate a genetic pathway shared with male pattern baldness, representing the first evidence for shared disease biology in men, and shared genetics with depression, previously postulated based only on observational studies.

## Introduction

Polycystic ovary syndrome (PCOS) is the most common endocrine disorder in reproductive aged women, with a complex pattern of inheritance [1–5]. Two different diagnostic criteria based on expert opinion have been utilized: The National Institutes of Health (NIH) criteria require hyperandrogenism (HA) and ovulatory dysfunction (OD) [6] while the Rotterdam criteria include the presence of polycystic ovarian morphology (PCOM) and requires at least two of three traits to be present, resulting in four phenotypes (S1 Fig) [6,7]. PCOS by NIH criteria has a prevalence of ~7% in reproductive age women worldwide [8]; the use of the broader Rotterdam criteria increases this to 15–20% across different populations [9–11].

PCOS is commonly associated with insulin resistance, pancreatic beta cell dysfunction, obesity and type 2 diabetes (T2D). These metabolic abnormalities are most pronounced in women with the NIH phenotype [12]. In addition, the odds for moderate or severe depression and anxiety disorders are higher in women with PCOS [13]. However, the mechanisms behind the association between the reproductive, metabolic and psychiatric features of the syndrome remain largely unknown.

Genome-wide association studies (GWAS) in women of Han Chinese and European ancestry have reproducibly identified 16 loci [14–17]. The observed susceptibility loci in PCOS appeared to be shared between NIH criteria and self-reported diagnosis [17], which is particularly intriguing. Genetic analyses of causality (by Mendelian Randomization analysis) among women of European ancestry with self-reported PCOS suggested that body mass index (BMI), insulin resistance, age at menopause and sex hormone binding globulin contribute to disease pathogenesis [17].

We performed the largest GWAS meta-analysis of PCOS to date, in 10,074 cases and 103,164 controls of European ancestry diagnosed with PCOS according to the NIH (2,540 cases and 15,020 controls) or Rotterdam criteria (2,669 cases and 17,035 controls), or by self-reported diagnosis (5,184 cases and 82,759 controls) (Tables 1 and S1). We investigated whether there were differences in the genetic architecture across the diagnostic criteria, and whether there were distinctive susceptibility loci associated with the cardinal features of PCOS; HA, OD and PCOM. Further, we explored the genetic architecture with a range of phenotypes related to the biology of PCOS, including male-pattern balding [18–21].

**Table 1:** Characteristics of PCOS cases and controls from each cohort included in the meta-analysis.

Cohort	Subject Type	Number	Age (years)	BMI (kg/m2)	PCOS Definition	HA^(^^1^^)^ n(%)	OD n(%)	PCOM n(%)
**Rotterdam**	Cases**	1184	28.8 (4.8)	26.1 (6.3)	NIH (41%) & Rotterdam (100%)^(^^2^^)^	439 (37.0)	946 (79.8)	661 (55.8)
	Controls	5799	60.5 (7.9)	27.6 (4.7)	Population Based Rotterdam Study	NA	NA	NA
**UK (London/ Oxford)**	Cases**	670	32.1 (6.8)	28.2 (7.9)	NIH (33%) & Rotterdam (100%)^(^^2^^)^	455 (67.9)	537 (80.1)	383 (57.2)
	Controls	1379	45 (0)^§^	26.8 (5.5)	1958 British Birth Cohort	NA	NA	NA
**EGCUT**	Cases**	157	30.7 (8.2)	26.2 (6.7)	Rotterdam^(^^2^^)^	NA	NA	NA
	Controls	2807	31.5 (7.3)	23.1 (5.5)	Population Based	NA	NA	NA
**deCODE**	Cases**	658	41.3 (8.7)	30.1 (7.8)	NIH (56%) & Rotterdam (100%)^(^^2^^)^	644 (97.9)	380 (57.7)	507 (77.1)
	Controls	6774	49.0 (9.9)	25.1 (4.9)	Population Based	NA	NA	NA
**Chicago**	Cases*	984	28.6 (5.5)	35.9 (8.5)	NIH	984 (100)	984 (100)	NA
	Controls	2963	46.8 (15.2)	27.0 (7.4)	Population Based NUgene	NA	NA	NA
**Boston**	Cases*	485	28.4 (6.7)	30.8 (8.7)	NIH	485 (100)	485 (100)	441 (90.9)
	Controls	407	27.2 (6.5)	23.8 (4.1)	Screened controls^(^^3^^)^	0	0	177 (43.4)
**23andMe**	Cases***	5,184	45.1 (13.6)	29.2 (8.2)	Self report (defined by questionnaire)	NA	NA	NA
	Controls	82,759	51.1 (15.7)	26.1 (6.1)	No PCOS by self report (defined by questionnaire)	NA	NA	NA

(1) Clinical or Biochemical.

(2) Rotterdam diagnostic criteria include the NIH criteria. All subjects from the indicated cohorts were used in the Rotterdam analysis.

(3) Controls were screened for regular menses and no hyperandrogenism.

* PCOS diagnosis was based on NIH criteria,

** Rotterdam criteria, or

*** self report.

Results are reported as mean (SD) or a number (%).

Abbreviations: BMI: body mass index, NA: not available, HA: hyperandrogenism, OD: ovulatory dysfunction (<10 menses per year), PCOM: polycystic ovarian morphology.

^§^All subjects are from the British Birth Cohort (born in 1958).

## Results

**Fig 1:**
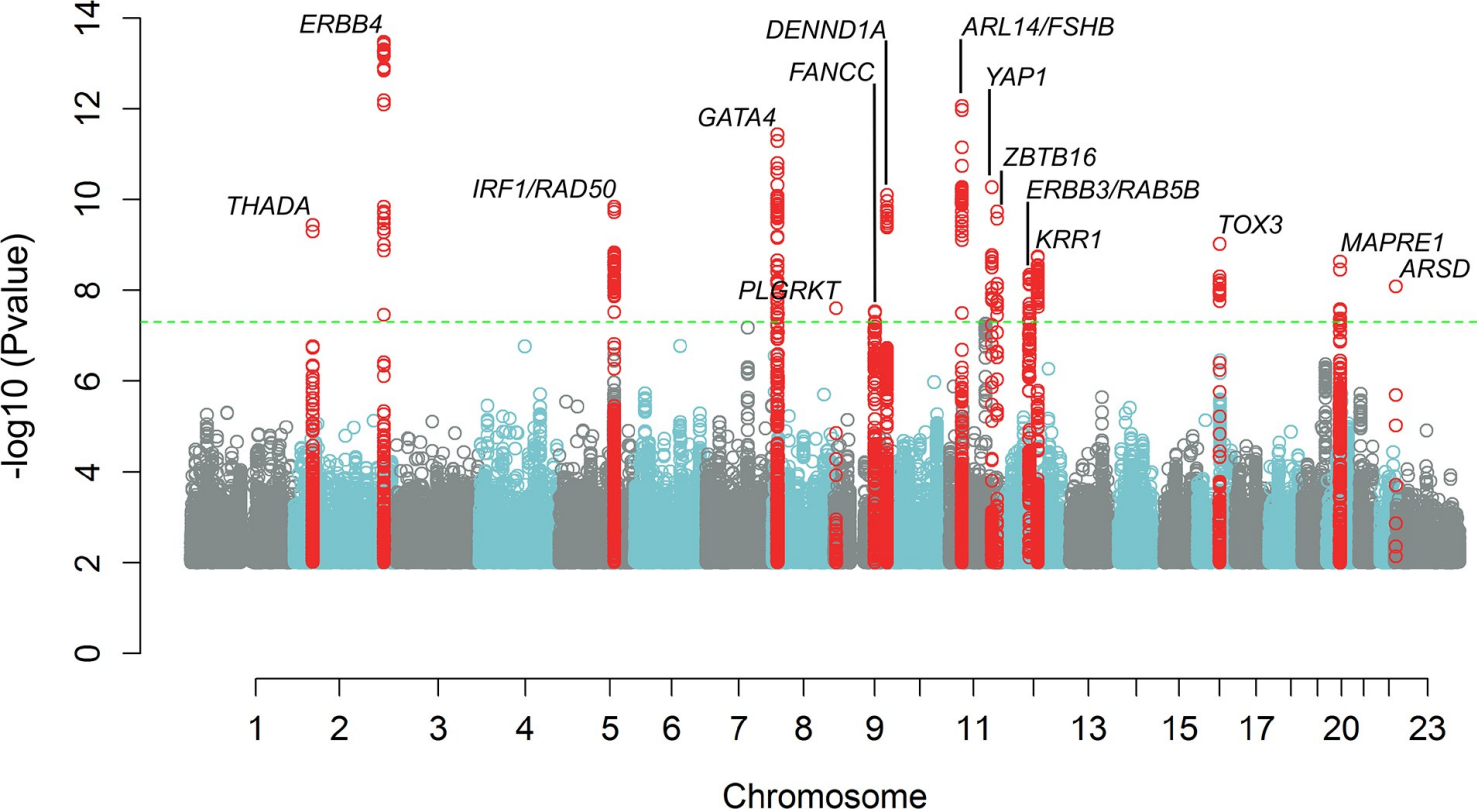
Manhattan plot showing results of meta-analysis for PCOS status, adjusting for age. The inverse log10 of the *p* value (-log10(*p*)) is plotted on the Y axis. The green dashed line designates the minimum *p* value for genome-wide significance (<5.0 x 10^−8^). Genome wide significant loci are denoted with a label showing the nearest gene to the index SNP at each locus. SNPs with *p* values ≤1.0x10^-2^ are not depicted.

**Fig 2:**
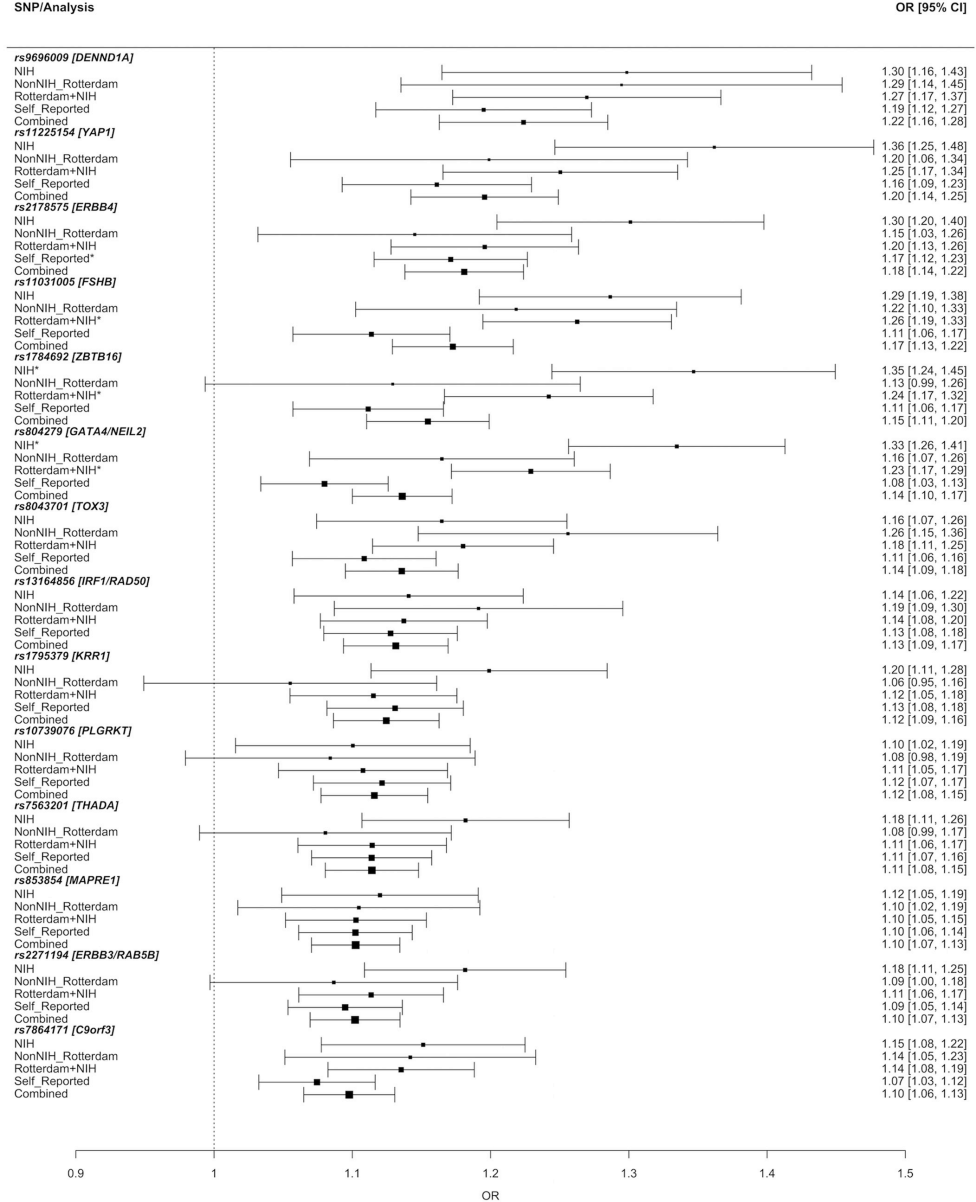
Odds ratio of polycystic ovary syndrome (PCOS) as a function of diagnostic criteria applied. The Y-axis specifies the diagnostic criteria and the X-axis indicates the odds ratio (OR) and 95% confidence intervals (CI) for PCOS (black circle and horizontal error bars). Data derived as follows: NIH = groups recruiting only NIH diagnostic criteria; NonNIH_Rotterdam = Rotterdam diagnostic criteria excluding the subset fulfilling NIH diagnostic criteria; Rotterdam+NIH = all groups except self-reported; self-reported = 23andMe; and combined = all groups. Specific OR’s [95% CI, 5% CI] are indicated on the right. rs804279 in the GATA4/NEIL2 locus demonstrates significant heterogeneity (Het P = 2.6x10^-5^). The * indicates statistically significant association for PCOS and the variant in that specific stratum.

We identified 14 genetic susceptibility loci associated with PCOS, adjusting for age, at the genome-wide significance level (P < 5.0 x 10^−8^) bringing the total number of PCOS associated loci to nineteen (Tables 2 and S2 and Fig 1). Three of these loci were novel associations (near *PLGRKT*, *ZBTB16* and *MAPRE1*, respectively; shown in bold in Table 2). Six of the 11 reported associations were previously observed in Han Chinese PCOS women [14,15]. Eight loci have been reported in European PCOS cohorts [16,17]. Obesity is commonly associated with PCOS and in most of the cohorts, cases were heavier than controls (Table 1). However, adjusting for both age and BMI did not identify any novel loci; and the 14 loci remained genome-wide significant. All variants demonstrated the same direction of effect across all phenotypes including NIH, non-NIH Rotterdam, and self-report (Fig 2 and S2 Table). Only one SNP near *GATA4/NEIL2* showed significant evidence of heterogeneity across the different diagnostic groups (rs804279, Het P = 2.6x10^-5^; Fig 2 and S3 Table). For this SNP, the largest effect was seen in NIH cases and the smallest in self-reported cases. Credible set analysis, which prioritises variants in a given locus with regards to being potentially causal, was able to reduce the plausible interval for the causal variant(s) at many loci (S4 Table). Of note, 95% of the signal at the *THADA* locus came from two SNPs. Examination of previously published genome-wide significant loci from Han Chinese PCOS [14,15] demonstrated that index variants from the *THADA*, *FSHR*, *C9orf3*, *YAP1* and *RAB5B* loci were significantly associated with PCOS after Bonferroni correction for multiple testing in our European ancestry subjects (S5 Table).

**Table 2:** The 14 genome-wide significant variants associated with PCOS in the meta-analysis.

Chr:Position^1^	rsID	Alleles^2^	EAF^3^	Beta	Odds Ratio (95% CI)^4^	Std. Error	Nearest Gene	P-value	Effective N^5^	Ref^6^
2:43561780	rs7563201	A/[G]	0.4507	-0.1081	0.90 (0.87–0.93)	0.0172	*THADA*	3.678e-10	17192	
2:213391766	rs2178575	G/[A]	0.1512	0.1663	1.18 (1.13–1.23)	0.0219	*ERBB4*	3.344e-14	17192	17
5:131813204	rs13164856	[T]/C	0.7291	0.1235	1.13 (1.09–1.18)	0.0193	*IRF1/RAD50*	1.453e-10	17192	17
8:11623889	rs804279	A/[T]	0.2616	0.1276	1.14 (1.10–1.18)	0.0184	*GATA4/NEIL2*	3.761e-12	16895	16
**9:5440589**	**rs10739076**	**C/[A]**	**0.3078**	**0.1097**	**1.12 (1.07–1.16)**	**0.0197**	***PLGRKT***	**2.510e-08**	**17192**	
9:97723266	rs7864171	G/[A]	0.4284	-0.0933	0.91 (0.88–0.94)	0.0168	*FANCC*	2.946e-08	17192	16
9:126619233	rs9696009	G/[A]	0.0679	0.202	1.22 (1.15–1.30)	0.0311	*DENND1A*	7.958e-11	17192	
11:30226356	rs11031005	[T]/C	0.8537	-0.1593	0.85 (0.82–0.89)	0.0223	*ARL14EP/FSHB*	8.664e-13	17192	16,17
11:102043240	rs11225154	G/[A]	0.0941	0.1787	1.20 (1.13–1.26)	0.0272	*YAP1*	5.438e-11	17192	17
**11:113949232**	**rs1784692**	**[A]/G**	**0.8237**	**0.1438**	**1.15 (1.10–1.14)**	**0.0226**	***ZBTB16***	**1.876e-10**	**17192**	
12:56477694	rs2271194	A/[T]	0.416	0.0971	1.10 (1.07–1.14)	0.0166	*ERBB3/RAB5B*	4.568e-09	17192	17
12:75941042	rs1795379	C/[T]	0.2398	-0.1174	0.89 (0.86–0.92	0.0195	*KRR1*	1.808e-09	17192	17
16:52375777	rs8043701	[A]/T	0.815	-0.1273	0.88 (0.85–0.92)	0.0208	*TOX3*	9.610e-10	17192	
**20:31420757**	**rs853854**	**A/[T]**	**.4989**	**-.0975**	**0.91 (0.88–0.94)**	**0.0163**	***MAPRE1***	**2.358e-09**	**17192**	

^1^Chr—Chromosome:Position (bp) in hg19;

^2^Alleles are shown as Major/Minor by allele frequency in 1000G EUR cohort, with the effect allele shown within [];

^3^Effect allele frequency;

^4^95% Confidence Interval of the Odds Ratio;

^5^Effective N—effective sample size;

^6^Ref = Reference.

Loci previously identified in GWAS studies of European ancestry are referenced. Novel associations with PCOS not previously reported are shown in bold. EAF = Effect Allele Frequency.

We assessed the association of the PCOS susceptibility variants identified in the GWAS meta-analysis with the PCOS related traits: HA, OD, PCOM, testosterone, FSH and LH levels, and ovarian volume in PCOS cases (Tables 3 and S6 and S2 Fig). We found four variants associated with HA, eight variants associated with PCOM and nine variants associated with OD. Of the eight loci associated with PCOM, seven were also associated with OD. Three of the four loci associated with HA were also associated with OD and PCOM. Two additional loci were associated with OD alone, one of which was the locus near *FSHB* (S6 Table). This locus was also associated with LH and FSH levels. There was a single PCOS locus near *IRF1/RAD50* associated with testosterone levels (S6 Table). We repeated this analysis with susceptibility variants reported previously in Han Chinese PCOS cohorts [14,15]. In this analysis, there was one association with HA (near *DENND1A*), three with PCOM and three with OD (S2 Fig and S5 Table). A limitation of these analyses is the variable sample size across the phenotypes analysed. Additionally, the known referral bias for the more severely affected NIH phenotype (patients having both OD and HA) may result in more PCOS diagnoses than the other criteria [22], and may have contributed to the number of associations between the identified PCOS risk loci and these phenotypes.

**Table 3:** Association of PCOS GWAS meta-analysis susceptibility variants and PCOS related traits.

Chr:Position	rsID	Gene	Ref. allele	Other allele	Hyperandrogenism			PCOM		OD	
					EAF	Beta	P-value	Beta	P-value	Beta	P-value
2:213391766	rs2178575	ERBB4*	G	A	0.83	-0.126	4.3E-03	**-0.24**	**1.4E-05**	**-0.23**	**1.2E-11**
2:43561780	rs7563201	THADA*^†^	G	A	0.56	0.061	8.0E-02	**0.16**	**3.7E-04**	0.08	1.5E-03
5:131813204	rs13164856	IRF1/RAD50*	T	C	0.73	0.092	1.8E-02	0.16	1.4E-03	0.08	5.6E-03
8:11623889	rs804279	GATA4/NEIL2*	A	T	0.27	0.126	8.7E-04	**0.22**	**1.5E-06**	**0.16**	**9.9E-09**
9:126619233	rs9696009	*DENND1A*^*†*^	G	A	0.94	**-0.330**	**2.9E-07**	**-0.32**	**4.0E-05**	**-0.36**	**4.4E-15**
9:5440589	rs10739076	*PLGRKT*	A	C	0.30	0.026	5.3E-01	0.10	5.9E-02	0.00	8.9E-01
9:97723266	rs7864171	C9orf3*^†^	G	A	0.60	**0.124**	**3.8E-04**	**0.19**	**1.3E-05**	**0.10**	**2.3E-04**
11:30226356	rs11031005	ARL14EP/FSHB*	T	C	0.85	-0.079	8.2E-02	-0.18	1.3E-03	**-0.13**	**2.8E-04**
11:102043240	rs11225154	YAP1*^†^	G	A	0.91	-0.144	1.4E-02	**-0.24**	**3.5E-04**	**-0.23**	**5.7E-08**
11:113949232	rs1784692	*ZBTB16*	T	C	0.85	0.146	4.6E-03	**0.30**	**2.8E-06**	**0.21**	**6.6E-09**
12:75941042	rs1795379	KRR1*	T	C	0.24	-0.104	8.0E-02	-0.16	1.5E-03	**-0.11**	**1.8E-04**
12:56477694	rs2271194	*ERBB3/RAB5B*^*†*^	A	T	0.42	**0.126**	**2.7E-04**	**0.17**	**7.9E-05**	**0.13**	**1.4E-06**
16:52375777	rs8043701	*TOX3*^*†*^	A	T	0.82	**-0.166**	**1.4E-04**	-0.17	1.5E-03	-0.08	9.2E-03
20:31420757	rs853854	*MAPRE1*	T	A	0.50	0.111	9.8E-04	0.10	2.1E-02	0.05	3.8E-02

Significant associations are highlighted in bold. Variant previously reported as a PCOS risk variant in

*European or

^†^Han Chinese populations.

In the analyses looking at the weighted genetic risk score in the Rotterdam cohort, we observed an increase in the risk for PCOS (S3 Fig). Compared to individuals in the third quintile (reference group), individuals in the top 5th quintile of risk score have an OR of 1.9 (1.4–2.5; 95% CI) for PCOS based on NIH criteria and an OR of 2.1 (1.7–2.5; 95% CI) for Rotterdam criteria based PCOS. Of the associations, only the effect estimate for the Rotterdam criteria was significant, possibly due to the smaller size available with cases diagnosed according to the NIH criteria. When looking at the area under the ROC curves at SNPs with different P-value thresholds, we found a maximum AUC of 0.54 using SNPs with a P-value < 5x10^-6^ for both diagnostic criteria. While this is significantly better than chance, it is unlikely that a risk score generated from the variants discovered to date would represent a clinically relevant tool.

LD score regression analysis revealed genetic correlations with childhood obesity, fasting insulin, T2D, HDL, menarche timing, triglyceride levels, cardiovascular diseases and depression (Table 4) suggesting that there is shared genetic architecture and biology between these phenotypes and PCOS. There were no genetic correlations with menopause timing or male pattern balding. Mendelian randomization suggested that there was a causal role for BMI, fasting insulin and depression pathways (Table 5). Interestingly, while there was no genetic correlation detected for male pattern balding or menopause timing with PCOS, the Mendelian randomization analyses were significant. The difference in the genetic correlation compared to the Mendelian randomization result suggests that there may be a small number of key biological process that are common between the phenotypes, and that the common genetic causal variants are limited only to the variants shared by the subset of key biological processes. The importance of BMI pathways on reproductive phenotypes was further demonstrated by the attenuation of significance of Mendelian randomization analysis for age-at-menarche when BMI-associated variants were excluded from the analysis.

**Table 4:** LD Score regression results using the LDSC method.

Phenotype	Genetic Correlation	SE	Z	P-value
Body mass index	0.34	0.039	8.60	8.21×10^−18^
Childhood obesity	0.34	0.066	5.17	2.40×10^−7^
Fasting insulin levels	0.44	0.087	5.01	5.33×10^−7^
Type 2 diabetes	0.31	0.068	4.47	7.84×10^−6^
High-density lipoprotein levels	-0.23	0.059	-3.96	7.40×10^−5^
Menarche	-0.16	0.042	-3.76	1.71×10^−4^
Triglyceride levels	0.19	0.052	3.61	3.05×10^−4^
Coronary artery disease	0.23	0.069	3.32	8.86×10^−4^
Depression	0.205	0.0582	3.5203	0.0004
Menopause	-0.014	0.0183	-0.762	0.4461
Male pattern balding	0.0149	0.0168	0.8861	0.3756

**Table 5:** Mendelian randomization using an inverse weighted variant method.

Potential Risk factor	IVW method^1^			MR-EGGER intercept p-value^2^
	Beta	SE	P-value	
Body mass index	0.72	0.072	1.56 x 10^−23^	0.13
Fasting insulin levels*	0.03	0.007	1.73 x 10^−5^	0.06
Male pattern balding	0.05	0.017	0.0034	0.93
Menopause	0.1	0.022	1.31 x 10^−5^	0.39
Depression	0.77	0.213	0.00029	0.64

*Loci used were initially reported in an analysis of fasting insulin adjusted for BMI.

^1^IVW = inverse weighted variant,

^2^Mendelian Randomization (MR)-Egger intercept p values were not significant. Therefore, MR-Egger results are not presented.

## Discussion

We found 14 independent loci significantly associated with the risk for PCOS, including three novel loci. The 11 previously reported loci implicated neuroendocrine and metabolic pathways that may contribute to PCOS (1.1 Note in S1 Data). Two of the novel loci contain potential endocrine related candidate genes. The locus harbouring rs10739076 contains several interesting candidate genes; *PLGRKT*, a plasminogen receptor and several genes in the insulin superfamily; *INSL6*, *INSL4* and *RLN1*, *RLN2* which are endocrine hormones secreted by the ovary and testis and are suspected to impact follicle growth and ovulation [23]. *ZBTB16* (also known as *PLZF*) has been marked as an androgen-responsive gene with anti-proliferative activity in prostate cancer cells [24]. *PLZF* activates *GATA4* gene transcription and mediates cardiac hypertrophic signalling from the angiotensin II receptor 2 [25]. Furthermore, *PLZF* is upregulated during adipocyte differentiation *in vitro* [26] and is involved in control of early stages of spermatogenesis [27] and endometrial stromal cell decidualization [28]. The third novel locus harbours a metabolic candidate gene; *MAPRE1* (interacts with the low-density lipoprotein receptor related protein 1 (LRP1), which controls adipogenesis [29] and may additionally mediate ovarian angiogenesis and follicle development [30] (1.2 Note in S1 Data). Thus, all the new loci contain genes plausibly linked to both the metabolic and reproductive features of PCOS.

We found that there was no significant difference in the association with case status for the majority of the PCOS-susceptibility loci by diagnostic criteria. All susceptibility variants demonstrated the same direction of effect for the NIH phenotype, non-NIH Rotterdam phenotype and self-report, with only one variant demonstrating significant heterogeneity among the groups. It is of considerable interest that the cohort of research participants from the personal genetics company 23andMe, Inc., identified by self-report, had similar risks to the other cohorts where the diagnosis was clinically confirmed. Our findings suggest that the genetic architecture of these PCOS definitions does not differ for common susceptibility variants. Only one locus, *GATA4/NEIL2* (rs804279), was significantly different across diagnostic criteria: most strongly associated in NIH compared to the Rotterdam phenotype and self-reported cases. Deletion of *GATA4* results in abnormal responses to exogenous gonadotropins and impaired fertility in mice [31]. The locus also encompasses the promoter region of *FDFT1*, the first enzyme in the cholesterol biosynthesis pathway [32], which is the substrate for testosterone synthesis, and is associated with non-alcoholic fatty liver disease [33]. The major difference between the NIH phenotype and the additional Rotterdam phenotypes is metabolic risk; the NIH phenotype is associated with more severe insulin resistance [34]. rs804279 does not show association with any of the metabolic phenotypes in the T2D diabetes knowledge portal {Type 2 Diabetes Knowledge Portal. type2diabetesgenetics.org. 2015 Feb 1; http://www.type2diabetesgenetics.org/variantInfo/variantInfo/rs804279} so it may represent a PCOS-specific susceptibility locus.

The significant association of PCOS GWAS meta-analysis susceptibility variants with the cardinal PCOS related traits OD, HA and PCOM further strengthened the hypothesis that specific variants may confer risk for PCOS through distinct mechanisms. Three variants at the *C9orf3*, *DENND1A*, and *RAB5B* were associated with all PCOS related traits. The findings were consistent with the Han Chinese *DENND1A* variant association with HA, as suggested previously [35]. Thus, these loci, along with *GATA4/NEIL2* (as discussed above) may help identify pathways that link specific PCOS related traits with greater metabolic risk. In contrast, the variants at the *ERBB4*, *YAP1*, and *ZBTB16* loci were strongly associated with OD and PCOM, and therefore, might be more important for links to menstrual cycle regularity and fertility. In addition, the *FSHB* variant was associated with the levels of FSH and LH [16,17], suggesting that it may act by affecting gonadotropin levels. This variant maps 2kb upstream from open chromatin (identified by DNase-Seq) and an enhancer (identified by peaks for both H3K27Ac and H3K4me1) in a lymphoblastoid cell line from ENCODE, indicating a potential role for a regulatory element ~25kb upstream from the *FSHB* promoter. Furthermore, the association between the *IRF1/RAD50* variant and testosterone levels may indicate a regulatory role in testosterone production.

Of note, results of the follow-up analysis show a high level of shared biology between PCOS and a range of metabolic outcomes consistent with the previous findings [17]. In particular, there is genetic evidence for increased BMI as a risk factor for PCOS. There is also genetic evidence that fasting insulin might be an independent risk factor. This study also confirmed a causal association with the pathways that underlie menopause [17], suggesting that PCOS has shared aetiology with both classic metabolic and reproductive phenotypes. Furthermore, there was an apparent effect of depression-associated variants on the likelihood of PCOS, suggesting a role for psychological factors on hormonally related diseases. However, the links between PCOS and depression might be complicated by pathways that are also related to BMI, as BMI pathways are causal in both PCOS and depression [36]. In addition, male-pattern balding-associated variants showed strong effects on PCOS, suggesting that this might be a male manifestation of PCOS pathways, as has been previously suggested [18,20,21,37]. This observation may reflect the biology of hair follicle sensitivity to androgens, seen in androgenetic alopecia, a well-recognised feature of HA and PCOS [38,39]. The Mendelian randomization results for male-pattern balding and menopause are significant despite non-significant genetic correlation results, suggesting that the shared aetiology may be specific to only a few key pathways.

In conclusion, the genetic underpinnings of PCOS implicate neuroendocrine, metabolic and reproductive pathways in the pathogenesis of disease. Although specific phenotype stratified analyses are needed, genetic findings were consistent across the diagnostic criteria for all but one susceptibility locus, suggesting a common genetic architecture underlying the different phenotypes. There was genetic evidence for shared biologic pathways between PCOS and a number of metabolic disorders, menopause, depression and male-pattern balding, a putative male phenotype. Our findings demonstrate the extensive power of genetic and genomic approaches to elucidate the pathophysiology of PCOS.

## Methods

### Ethics statement

All research involving human participants has been approved by the authors' Institutional Review Board (IRB) or an equivalent committee, and all clinical investigation was conducted according to the principles expressed in the Declaration of Helsinki. Written informed consent was obtained from all participants. The Boston cohort was approved by the Partners IRB (# 2002P001924) and the University of Utah IRB (IRB_00076659). The deCODE cohort was approved by the National Bioethics Committee of Iceland (VSN 03–007), which was conducted in agreement with conditions issued by the Data Protection Authority of Iceland. Personal identities of the participants’ data and biological samples were encrypted by a third-party system (Identity Protection System), approved and monitored by the Data Protection Authority. The UK cohort was approved by the Parkside Health Authority (Now—NHS Health Research Authority, NRES Committee—West London & GTAC, UK, London, UK) under EC2359 "The Molecular Genetics of Polycystic Ovaries." The Rotterdam PCOS cohort, the COLA study, was approved by institutional review board (Medical Ethics Committee) of the Erasmus Medical Center (04–263). Controls from the Rotterdam Study were approved by the Medical Ethics Committee of the Erasmus MC (registration number MEC 02.1015) and by the Dutch Ministry of Health, Welfare and Sport (Population Screening Act WBO, license number 1071272-159521-PG). The Rotterdam Study Personal Registration Data collection is filed with the Erasmus MC Data Protection Officer under registration number EMC1712001. The Rotterdam Study has been entered into the Netherlands National Trial Register (NTR; www.trialregister.nl) and into the WHO International Clinical Trials Registry Platform (ICTRP; www.who.int/ictrp/network/primary/en/) under shared catalogue number NTR6831. The Chicago PCOS cohort was approved by the Northwestern IRB (#STU00008096). The control subjects from the NUgene study were approved by the Northwestern IRB (# STU00010003). The Estonia cohort was approved by the Research Ethics Committee of the University of Tartu approved the study (198T-18). The Twins UK study was approved by the St Thomas' Hospital Research Ethics Committee (EC04/015). The Nurses' Health Study (NHS I and II) was approved by the Partners Human Research Committee (#1999-P-011114).

### Subjects

The meta-analysis included 10,074 cases and 103,164 controls from seven cohorts of European descent. For the analysis of PCOS related traits three additional cohorts, the Northern Finnish Birth Cohort (NFB66) [40], Twins UK [41] and the Nurses’ Health Study (NHS) [42] were included. Cases were diagnosed with PCOS based on NIH or Rotterdam Criteria or by self-report. The NIH criteria require the presence of both OD and clinical and/or biochemical HA for a diagnosis of PCOS [6]. The Rotterdam criteria require two out of three features 1) OD defined by oligo- or amenorrhea (chronic menstrual cycle interval >35 days in all cohorts), 2) clinical and/or biochemical hyperandrogenism (HA) and/or 3) PCOM for a diagnosis of PCOS [7]. Non-NIH Rotterdam was defined by OD and PCOM or clinical and/or biochemical hyperandrogenism (HA) and PCOM. Self-reported female cases from research participants in the 23andMe, Inc. (Mountain View, CA, USA) cohort either responded “yes” to the question “Have you ever been diagnosed with polycystic ovary syndrome?” or indicated a diagnosis of PCOS when asked about fertility (“Have you ever been diagnosed with PCOS?” or “What was your diagnosis? Please check all that apply.” Answer = PCOS), hair loss in men or women (“Have you been diagnosed with any of the following? Please check all that apply.” Answer = PCOS) or research question (“Have you ever been diagnosed with PCOS?”) [17]. 23andMe controls were female, only.

HA was defined as hirsutism and quantified by the Ferriman-Gallwey (FG) score. The FG score assesses terminal hair growth in a male pattern in females, and a score above the upper limit of normal controls (>8) is considered hirsutism [43]. Hyperandrogenemia was defined as testosterone, androstenedione or DHEAS greater than the 95% confidence limits in control subjects in the individual population. OD was defined as cycle interval <21 or >35 days [44]. PCOM was defined as 12 or more follicles of 2–9 mm in at least one ovary or an ovarian volume >10 mL [7]. The quantitative PCOS traits included levels of total testosterone (T), follicle-stimulating hormone (FSH), and luteinizing hormone (LH) and ovarian volume (S1 Table). An overview of the cohorts, diagnostic criteria and number of subjects included in each subphenotype or trait analysis are summarized in Tables 1 and S1.

### Data collection and quality control

Each study provided summary results of genetic per-variant estimates produced in either case-control or trait association analyses. Adjustment for principle components was performed at the study level. The collected files underwent quality control (QC) by two independent analysts using the EasyQC pipeline [45]. Variants were excluded based on minor allele frequency (MAF) < 1%, imputation quality (R^2^) < 0.3 or info < 0.4 for MACH and IMPUTE2 respectively [46,47]. Per-cohort QC results from EasyQC are shown (S7 Table), and allele frequency spectrum for each cohort, and the combined cohort after meta-analysis is shown (S4 Fig).

### Meta-analysis of PCOS status and PCOS related traits

The per-variant estimates collected from the summary statistics of contributing studies were meta-analysed using a fixed-effect, inverse-weighted-variance meta-analysis that employed either GWAMA [48] or METAL [49]. In addition to the overall meta-analysis, we performed meta-analyses for studies with available data for the separate PCOS diagnostic criteria: NIH, non-NIH Rotterdam [7] and self-report [17], as well as for the PCOS related traits of HA, OD and PCOM. The meta-analysis of PCOS status was performed using two models; (1) age-adjusted, (2) age and BMI-adjusted, given the high prevalence of obesity in affected women that resulted in cases being significantly heavier than controls in most cohorts (Table 1).

We removed any variants that were not present in more than 50% of the effective sample size prior to combining with 23andMe as this was the largest cohort in the meta-analysis, providing approximately 51% of the PCOS cases and 80% of controls. We also removed any variants only present in one study. The meta-analysis of PCOS related traits was performed adjusting for age and BMI. Identified variants were annotated for insight into their biological function using ANNOVAR [50] to assign refGene gene information, SIFT score [51], PolyPhen2 scores [52], CADD scores [53], GERP scores [54] and SiPhy log odds [55].

### Comparison of PCOS diagnostic criteria

In order to compare different PCOS diagnostic criteria [(1) NIH, (2) non-NIH Rotterdam and (3) self-reported] included in the PCOS meta-analysis, an additional meta-analysis was performed to test for heterogeneity across these independent PCOS case groups. These three PCOS case groups were combined in an inverse variance weighted fixed meta-analysis and the heterogeneity statistics (Cochran’s Q and I^2^) were obtained using GWAMA [48]. Any variant with a statistically significant Cochran’s Q p-value (P<0.05/14 = 0.0036 corrected for multiple testing) and I^2^>70% were considered exhibiting heterogeneity across the PCOS case groups. Further analysis of the heterogeneity included comparison of the 95% confidence intervals for the direction of effect and overlaps.

### Identifying associations between PCOS Loci and PCOS related traits

In order to understand biology relevant to identified PCOS susceptibility, we assessed the association between index SNPs at each genome-wide-significant locus and the PCOS related traits HA, OD, PCOM as well as the quantitative traits testosterone, LH and FSH levels and ovarian volume. The threshold for significance in this analysis was p<4.5×10^−4^ (Bonferroni correction [0.05/(14 independent loci x 8 traits)].

### Identifying shared risk loci between European ancestry and Han Chinese PCOS

In order to identify shared risk loci between the previously reported GWAS in Han Chinese PCOS cases and our European ancestry cohort, 13 independent signals (represented by 15 SNPs) at 11 genome-wide significant loci reported by Chen *et al*. [14] and Shi *et al*. [15] were investigated for association in our meta-analyses of PCOS and PCOS related traits. The adjusted P-value for this analysis was <0.00048 (Bonferroni correction [0.05/(13 independent signals x 8 traits)]).

### Biologic function of genes in associated loci

Information on the biological function of the nearest gene (or genes, if variants were equidistant from more than one coding transcript and annotated as such by ANNOVAR [49] to the index SNP of each identified risk locus) was collected by performing a search of the Entrez Gene Database [56], and collecting the co-ordinates of the gene (genome build 37; hg19) as well as the cytogenetic location and the summary of the gene function. In addition to the EntrezGene Database queries, the gene symbol was used as a search term in the PubMed database [57], either alone or combined with the additional search term “PCOS” to identify relevant published literature in order to obtain information on putative biological function and involvement in the pathogenesis of PCOS (summarized in 1.1 Note in S1 Data).

### Weighted genetic risk score and prediction

One potential use of genetic risk scores is prediction of disease. The ability of genetic risk scores calculated from loci discovered in analysis of the different diagnostic criteria to discriminate cases from alternative criteria was measured. We constructed a weighted genetic risk score based on a meta-analysis excluding the Rotterdam Study subjects. The weighted genetic risk score was divided into quintiles and tested for association with PCOS in the Rotterdam cohort. The middle quintile was used as the reference and the odds for having PCOS based on both Rotterdam and NIH criteria was then calculated.

Additionally, the 23andMe results were used to select independent SNPs with cut-offs of *p*<5×10^−4^ to *p*<5×10^−8^. The Rotterdam cohort was then used to calculate risk scores and the area-under-the curve (AUC) for both NIH and Rotterdam diagnostic criteria. Analyses were performed using PLINK v1.9 and SPSS v21 (IBM Corp, Armonk, NY) [58].

### Linkage disequilibrium (LD) score regression

To assess the level of shared etiology between PCOS and related traits, we performed genetic correlation analysis using LD-score regression [59]. Publicly available genome-wide summary statistics for body mass index (BMI) [60], childhood obesity [61], fasting insulin levels (adjusted for BMI) [62], type 2 diabetes [63], high-density lipoprotein (HDL) levels [64], menarche timing [65], triglyceride levels [64], coronary artery disease [66], depression [36], menopause [17] and male pattern balding [67] were used to estimate the genome-wide genetic correlation with PCOS. The adjusted P-value for this analysis was p<0.0045 after a Bonferroni correction (0.05/11 traits).

### Mendelian randomization

Phenotypes of interest, both where there was evidence of shared genetic architecture and where there was previous evidence for genetic links, were assessed using Mendelian randomization methods. Mendelian randomization differs from LD score regression in that one phenotype is analysed as a potential causal factor for another. Mendelian randomization was performed using both inverse weighted variance and Egger’s regression methods [68], with inverse weighted methods being more powerful, but Egger’s methods being resistant to directional pleiotropy (where there are a set of SNPs that appear to have an alternative pathway of effect). We report here the results of the IVW methods as none of the analysis suggested that the MR-EGGERs results were more appropriate given that none of the EGGERs intercepts were significant (Table 5). In addition to the phenotypes implicated by the LD-score regression measures, male pattern balding has a strong biological rationale and was therefore included. The genetic score for childhood obesity substantially overlaps with the score for adult BMI (such that the INSIDE violation—where the effect of SNPs on a confounding factor scales with that on the trait of interest—of Mendelian randomization would likely occur [69], so only a score for BMI was used, with the proviso that this represents BMI across the whole of the life course after very early infancy. The SNPs for depression were drawn from the results of a more recent analysis, for which there was not, at time of analysis, publicly available genome-wide data.

### Credible sets

We defined a locus as mapping within 500kb of the lead SNP. For each locus, we first calculated the posterior probability, π_Cj_, that the jth variant is driving the association, given by:

$$\pi_{cj}=\frac{\Lambda_{j}}{\Sigma_{k}\Lambda_{k}}$$

where the summation is over all retained variants in the locus. In this expression, Λ_j_ is the approximate Bayes’ factor [70] for the jth variant, given by

$$\Lambda_{j}=\sqrt{\frac{V_{j}+\omega }{V_{j}}}exp\left[-\frac{\omega \beta^{2_{j}}}{2V_{j}\left(V_{j}+\omega \right)}\right]$$

where β_j_ and V_j_ denote the estimated allelic effect (log-OR) and corresponding variance from the meta-analysis. The parameter ω denotes the prior variance in allelic effects, taken here to be 0.04 [70]. The 99% credible set [71] for each signal was then constructed by: (i) ranking all variants according to their Bayes’ factor, Λ_j_; and (ii) including ranked variants until their cumulative posterior probability of driving the association attained or exceeded 0.99.

## Supporting information

S1 Data Supplementary results suggestive evidence of a 15th signal, rs151212108, near *ARSD* on the X chromosome and literature lookup of genes at PCOS risk loci. (DOCX)

S1 Table Cohorts contributing polycystic ovary syndrome (PCOS) cases, PCOS phenotypes, laboratory data and controls. (XLSX)

S2 Table All PCOS meta-analysis, PCOS meta-analysis without self-report, NIH, non-NIH Rotterdam and self-report meta-analysis results. (XLSX)

S3 Table Heterogeneity analysis for NIH, non-NIH Rotterdam and self-report cohorts. (XLSX)

S4 Table Fine-mapping of PCOS risk loci identified in the meta-analysis to narrow candidate causal variants. (XLSX)

S5 Table Look-up of previously published PCOS risk variants in Han Chinese cohorts with PCOS GWAS meta-analysis and PCOS related traits (HA, OD, PCOM, T, FSH, LH and ovarian volume). (XLSX)

S6 Table All PCOS meta-analysis results and look-up of PCOS GWAS meta-analysis susceptibility variants with PCOS related traits (HA, OD, PCOM, T, FSH, LH and ovarian volume). (XLSX)

S7 Table Number of SNPs removed from each cohort after application of easy QC after application of each filter. (XLSX)

S1 Fig Diagnostic criteria of PCOS results in four distinct PCOS phenotypes. (DOCX)

S2 Fig Cluster plots showing relationships between PCOS loci and related traits. (DOCX)

S3 Fig Weighted genetic risk score to predict odds of PCOS based on either Rotterdam or NIH criteria. (DOCX)

S4 Fig Allele frequency spectrum from each cohort and the combined cohort for meta-analysis. (DOCX)
