# Correction: Large-scale genome-wide meta-analysis of polycystic ovary syndrome suggests shared genetic architecture for different diagnosis criteria

**DOI:** 10.1371/journal.pgen.1008517

**Published:** 2019-12-05

**Authors:** Felix Day, Tugce Karaderi, Michelle R. Jones, Cindy Meun, Chunyan He, Alex Drong, Peter Kraft, Nan Lin, Hongyan Huang, Linda Broer, Reedik Magi, Richa Saxena, Triin Laisk, Margrit Urbanek, M. Geoffrey Hayes, Gudmar Thorleifsson, Juan Fernandez-Tajes, Anubha Mahajan, Benjamin H. Mullin, Bronwyn G. A. Stuckey, Timothy D. Spector, Scott G. Wilson, Mark O. Goodarzi, Lea Davis, Barbara Obermayer-Pietsch, André G. Uitterlinden, Verneri Anttila, Benjamin M. Neale, Marjo-Riitta Jarvelin, Bart Fauser, Irina Kowalska, Jenny A. Visser, Marianne Andersen, Ken Ong, Elisabet Stener-Victorin, David Ehrmann, Richard S. Legro, Andres Salumets, Mark I. McCarthy, Laure Morin-Papunen, Unnur Thorsteinsdottir, Kari Stefansson, the 23andMe Research Team, Unnur Styrkarsdottir, John R. B. Perry, Andrea Dunaif, Joop Laven, Steve Franks, Cecilia M. Lindgren, Corrine K. Welt

In the Data Availability Statement, the URL does not fully match the data described. The URL should be: https://doi.org/10.17863/CAM.36024. The correct Data Availability Statement is: Summary statistic GWAS meta-analysis results for the combined dataset excluding 23andMe are available at https://doi.org/10.17863/CAM.36024. The most significant 10,000 SNPs for the meta-analysis including 23andMe are available at https://doi.org/10.17863/CAM.36024.

There is an error in Table 2. The square brackets that indicate the effect allele are shown in error. Please see the correct Table 2 here.

**Table 2:** The 14 genome-wide significant variants associated with PCOS in the meta-analysis.

Chr. Position^1^	rsID	EA^2^	OA^3^	EAF^4^	Beta	OR^5^	95% CI^6^	Std. Error	Nearest Gene	P-value	Effective N^7^	Ref^8^
2:43561780	rs7563201	A	G	0.451	-0.108	0.90	(0.86±0.92)	0.0172	*THADA*	3.68e-10	17192	
2:213391766	rs2178575	A	G	0.151	0.166	1.18	(1.13±1.23)	0.0219	*ERBB4*	3.34e-14	17192	17
5:131813204	rs13164856	T	C	0.729	0.124	1.13	(1.08±1.17)	0.0193	*IRF1/ RAD50*	1.45e-10	17192	17
8:11623889	rs804279	A	T	0.262	0.128	1.14	(1.09±1.17)	0.0184	*GATA4/ NEIL2*	3.76e-12	16895	16
9:5440589	**rs10739076**	**A**	**C**	**0.308**	**0.110**	**1.12**	**(1.07±1.15)**	**0.0197**	***PLGRKT***	**2.51e-08**	**17192**	** **
9:97723266	rs7864171	A	G	0.428	-0.093	0.91	(0.88±0.94)	0.0168	*FANCC*	2.95e-08	17192	16
9:126619233	rs9696009	A	G	0.068	0.202	1.22	(1.15±1.30)	0.0311	*DENND1A*	7.96e-11	17192	
11:30226356	rs11031005	T	C	0.854	-0.159	0.85	(0.81±0.89)	0.0223	*ARL14EP/ FSHB*	8.66e-13	17192	16,17
11:102043240	rs11225154	A	G	0.094	0.179	1.20	(1.13±1.26)	0.0272	*YAP1*	5.44e-11	17192	17
11:113949232	**rs1784692**	**T**	**C**	**0.824**	**0.144**	**1.15**	**(1.10±1.20)**	**0.0226**	***ZBTB16***	**1.88e-10**	**17192**	
12:56477694	rs2271194	A	T	0.416	0.097	1.10	(1.06±1.13)	0.0166	*ERBB3/ RAB5B*	4.57e-09	17192	17
12:75941042	rs1795379	T	C	0.240	-0.117	0.89	(0.85±0.92)	0.0195	*KRR1*	1.81e-09	17192	17
16:52375777	rs8043701	A	T	0.815	-0.127	0.88	(0.84±0.91)	0.0208	*TOX3*	9.61e-10	17192	
20:31420757	**rs853854**	**A**	**T**	**0.499**	**-0.098**	**0.91**	**(0.87±0.93)**	**0.0163**	***MAPRE1***	**2.36E-09**	**17192**	

1. Chromosome and position in hg19.

2. EA–Effect allele.

3. OA–Other allele.

4. EAF—Effect allele frequency.

5. OR–Odds Ratio.

6. 95% CI– 95 percent confidence interval.

7. Effective N–effective sample size.

8. Ref.–reference.

S2 Table is a duplicate of S6 Table. Please view the correct S2 Table below.

## Supporting information

S2 Table All PCOS meta-analysis, PCOS meta-analysis without self-report, NIH, non-NIH Rotterdam and self-report meta-analysis results. (XLSX)
