# Supplementary material for: Large-scale genome-wide meta-analysis of polycystic ovary syndrome suggests shared genetic architecture for different diagnosis criteria
Source: PLoS Genet. 2018 Dec 19;14(12):e1007813. doi: 10.1371/journal.pgen.1007813 (PMC6300389; doi:10.1371/journal.pgen.1007813)
Supplement: S1 Data — (DOCX) [file pgen.1007813.s001.docx]

***S1 Data. Supplementary Materials.***

##

## **SUPPLEMENTARY NOTES**

**1.1. Supplementary Results.**

In addition to these 14 significant loci, there was suggestive evidence of a 15th signal, rs151212108, near *ARSD* on the X chromosome. This SNP shows a relatively large effect size (OR:1.72, CI:1.43-2.07, P=8.35x10^-9^). However, the SNP had low sample number (overall minor allele frequency=0.0765) with poor imputation quality and it was present in only three studies; Oxford, deCODE, and Chicago. Further, the signal showed nominally significant heterogeneity (P =0.028) in the direction of effect estimates between Oxford, where the effect allele had a protective effect, and deCODE and Chicago, where the effect allele increased risk of PCOS. Thus, this signal was less robust than our other signals and will require further confirmation. Accordingly, we have not included this locus in downstream analyses. A detailed review of genes within reported loci is included in the Supplementary Notes (Section 1.2).

**1.2 Literature Lookup of genes at PCOS risk loci.** Summary of published literature on gene function of PCOS susceptibility loci.

**1.** **THADA (Thyroid Adenoma Associated): Located at 2p21 (Chr 2: 43561780-43561780).** Encodes a transcript of largely unknown function. THADA encodes thyroid adenoma-associated protein, which is expressed in pancreas, adrenal medulla, thyroid, adrenal cortex, testis, thymus, small intestine, and stomach.^71^ This gene has been identified in GWAS for gestational weight gain, inflammatory bowel disease and PCOS and more specifically with the phenotype trait PCOM.^14,71-74^ THADA has been associated with endocrine and metabolic disturbances commonly found in PCOS, such as increased LH, testosterone and LDL levels and T2D.^75^ Proposed to modify PCOS risk through metabolic mechanisms.^76^

**2. ERBB4 (erb-b2 receptor tyrosine kinase 4; also known as HER4): Located at 2q33.3-q34.** Fourth member of the EGFR (epidermal growth factor *receptor*) family and the Tyr protein kinase family (USCS, GeneNetwork, RefSeq). Participates in the YAP/Hippo pathway, which regulates cell proliferation, differentiation and apoptosis and has been associated with the size of the primordial follicle pool in mice, female reproductive capacity in Drosophila, and it is hypothesized that disruption of Hippo signaling can promote follicle growth.^77-81^ Tyrosine-protein kinase plays an essential role as cell surface receptor for neuregulins and EGF family members and regulates development of the heart, the central nervous system and the mammary gland.^82^ HER4 is characterized by anti-proliferative and pro-apoptotic activity, is co-expressed in 90% of ER positive breast tumors. Proposed to modify PCOS risk through metabolic mechanisms.^76^ Suggested to have a pathogenic role in cystogenesis in polycystic kidney disease.^83^

**3.** **IRF1 (interferon regulatory factor 1): Located at 5q31.1 (Chr5: 131813204-131813204).** Belongs to the interferon regulatory transcription factor (IRF) family. Activates the transcription of interferons alpha and beta, and genes induced by interferons alpha, beta and gamma (USCS, GeneNetwork, NCBI gene database). IRF1 displays a functional diversity in the regulation of cellular responses including host response to viral and bacterial infections, inflammation, and cell proliferation and differentiation, regulation of the cell cycle and induction of growth arrest and programmed cell death following DNA damage (UniProtKB). Acts as a tumor suppressor and plays a role not only in antagonism of tumor cell growth but also in stimulating an immune response against tumor cells.^84^ It has been shown in fathead minnow that IRF1 may function as early molecular switch to control phenotypic changes in ovary tissue architecture and function in response to androgen or antiandrogen exposure.^85^

**4. RAD50 (RAD50 homolog): Located at 5q31 (Chr5: 131892616-131980313).** Rad50, a protein involved in DNA double-strand break repair.^82^ This protein forms a complex with MRE11 and NBS1. The protein complex binds to DNA and displays numerous enzymatic activities that are required for non-homologous joining of DNA ends, and is important for DNA double-strand break repair,^86^ telomere maintenance, and meiotic recombination.^87^ Knockout studies of the mouse homolog suggest this gene is essential for cell growth and viability.^88^

**5.** **GATA4 (GATA binding protein 4): Located at 8p23.1-p22 (Chr 8: 11623889-11623889).** GATA4 encodes a zinc-finger transcription factor that recognizes the GATA motif in the promoters of various genes (RefSeq). GATA4 is implicated in regulating granulosa cell differentiation, proliferation and function and is expressed in follicles, embryoid bodies and chorion of women with PCOS.^89^ Knockdown of GATA4 and GATA6 impairs folliculogenesis and induces infertility.^30,90^ The loss of GATA4 within the ovary results in impaired granulosa cell proliferation and theca cell recruitment.^91^ Knockdown of both genes affects expression of FSH receptor, LH receptor, inhibin α and β.^30,89^ In rats with reproductive and metabolic abnormalities similar to PCOS, GATA4 has been associated with the biosynthesis and metabolism of steroids.^92^ It is also proposed to modify PCOS risk through metabolic and inflammatory mechanisms.^76^

**6.** **PLGRKT (Plasminogen receptor, C-terminal lysine transmembrane protein): Located at 9p24.1 (Chr 9; 5440339-5440839).** PLGRKT encodes a plasminogen receptor involved in regulating macrophage migration and regulates catecholamine release.^93^ The region also includes genes for several members of the insulin superfamily (INSL6, INSL4, RLN1, RLN2), which have roles in spermatogenesis, follicle growth and ovulation.^22,94^

**7.** **FANCC (Fanconi anemia, complementation group C): Located at: 9q22.3 (Chr 9: 97723266-97723266).** Member of the Fanconi anemia complementation group which, amongst others, includes FANCD1 (BRCA2). Members of the Fanconi anemia complementation group are related by their assembly into a common nuclear protein complex (RefSeq). This gene encodes the protein for complementation group C. Fanconi anemia is a recessive repair deficiency disorder, characterized by cytogenetic instability, hypersensitivity to DNA crosslinking agents, chromosomal breakage and defective DNA repair (UCSC, GeneNetwork). FANCC is a DNA repair protein that may operate in a post replication repair or a cell cycle checkpoint function.^82^ May be implicated in interstrand DNA cross-link repair and in the maintenance of normal chromosome stability. Was recently shown to have a mitophagy function as well, and is required for clearance of damaged mitochondria.^95^

**8.** **C9orf3. Located at 9q22.32.** Has been previously associated with PCOS.^15^ However, the region also includes genes for two hormones that regulate gluconeogenesis (FBP1, FBP2), and for PTCH1, which is a receptor for hedgehog proteins. In mice, the hedgehog signaling has been shown to be important for ovarian follicle development and is also implicated in the proliferation and steroidogenesis of theca cells.^96^ This is supported by the association between rs4385527 in C9orf3 and anovulation, HA and polycystic ovarian morphology (PCOM).^73^

**9.** **DENND1A (DENN/MADD domain-containing protein 1A): Located at 9q33.3 (Chr 9: 126619233-126619233).** Member of the connecdenn family and functions as a guanine nucleotide exchange factor involved for the early endosomal small GTPase RAB35 (UCSC, RefSeq). Regulates clathrin-mediated endocytosis (a major mechanism for internalization of proteins and lipids) through RAB35 activation (USCS, RefSeq). DENND1A variant 2 (DENND1A.V2) protein and mRNA levels are increased in PCOS theca cells and play a key role in the hyperandrogenemia associated with PCOS.^97^ The *DENND1A* locus has also been associated with PCOM and elevated serum insulin levels in PCOS women.^73,75^ Some SNP’s in DENND1A have even been associated with endometrioid carcinoma. It has been suggested that *DENND1A, LHCGR, INSR*, and *RAB5B* form a hierarchical signalling network that can influence androgen synthesis.^98^

**10.** **ARL14EP (ADP-ribosylation factor-like 14 effector protein): Located at 11p14.1.** Encodes an effector protein, which interacts with ADP-ribosylation factor-like 14 [ARL14], beta-actin and actin-based motor protein myosin 1E. ARL14 controls the export of major histocompatibility class II molecules by connecting to the actin network via this effector protein (RefSeq).

**11.** **FSHB (Follicle stimulating hormone, beta polypeptide): Located at 11p14.1 (Chr 11; 30226356-30226356).** FSHB is a member of the pituitary glycoprotein hormone family and encodes the β-subunit of the follicle-stimulating hormone (FSH) (RefSeq). FSH regulates folliculogenesis. FSHB polymorphisms influence early follicular phase FSH concentrations and IVF treatment outcome.^99^ SNPs in the FSHB region are known to be associated with circulating FSH, LH and AMH levels but also with PCOS.^99-105^ Overexpression of FSHB could cause polycystic ovary syndrome in women, whereas inactivating mutations of the FSHB gene, encoding for the hormone's unique β-subunit, cause infertility by primary amenorrhea.^106,107^

**12.** **YAP1 (Yes-associated protein 1): Located at 11q13 (Chr 11; 102043240-102043240).** YAP1 is an effector protein in the Hippo pathway involved in development, growth, repair, and homeostasis (RefSeq). This pathway also plays a pivotal role in organ size control and tumor suppression by restricting proliferation and promoting apoptosis.^82^ Has been associated with the size of the primordial follicle pool in mice, female reproductive capacity in *Drosophila*, and it is hypothesized that disruption of Hippo signaling can promote follicle growth.^79^ The candidacy of YAP1 as a susceptibility gene for PCOS has been highlighted in several studies.^15,108,109^

**13.** **ZBTB16 (Zinc finger and BTB domain containing 16, also known as PLZF): Located at 11q23.1 (Chr 11; 113949232-113949232).** Member of the Krueppel C2H2-type zinc-finger protein family and encodes a zinc finger transcription factor that contains nine Kruppel-type zinc finger domains at the carboxyl terminus. This protein is located in the nucleus and is involved in cell cycle progression. The zinc finger protein has a pro-apoptotic and anti-proliferative activity and has been marked as an androgen-responsive gene with anti-proliferative activity in prostate cancer cells.^23^ PLZF binds to the GATA4 gene regulatory region and activates GATA4 transcription and mediates cardiac hypertrophic signaling from angiotensin II receptor 2.^24^ The loss of PLZF has been related to increased proliferation, invasiveness and motility, and resistance to apoptosis in different cancer cell types.^110^ PLZF is considered a tumor suppressor gene in various cell types and tissues. Up-regulated during adipocyte differentiation *in vitro.*^25^ Involved in control of early stages of spermatogenesis,^26^ and critical for endometrial stromal cell decidualization.^27^

**14.** **ERBB3 (erb-b2 receptor tyrosine kinase 3; also known as HER3): Located at 12q13.** A member of the EGFR family of receptor tyrosine kinases (RefSeq). The ERBB3 gene is a potential susceptibility locus for T1D and has also been associated with PCOS.^15,111^ ERBB4 together with ERBB3-binding protein 1 may modulate the protein cascade that leads to differentiation of ovarian somatic cells. ERBB3 interacts with the YAP protein in the Hippo pathway and is implicated in ovarian cell tumors.^112^ The same region also includes RAB5B and a SNP in this region has been associated with response to glycose stimulation.^113^

**15.** **RAB5B (Member of the RAS oncogene family): Located at 12q13.** Member of the RAS oncogene family. RAB5B is an isoform of RAB5, a member of the small G protein family. Rab5 regulates fusion and motility of early endosomes, and is a marker of the early endosome compartment.^114^ Endogenous Rab5B may work in conjunction or in sequence with Rab5A to facilitate the trafficking of EGFR.^115^ RAB5b has previously been identified in PCOS in women of Han Chinese and European descent.^109^ A variant near this gene has been associated with insulin and glucose levels.^113^ It has been suggested that *DENND1A, LHCGR, INSR*, and *RAB5B* form a hierarchical signaling network that can influence androgen synthesis.^98^ Proposed to modify PCOS risk through metabolic mechanisms.^76^ RAB5B shows lower expression levels in adipose tissue from PCOS women compared to healthy controls.^116^

**16.** **KRR1 (KRR1, small subunit (SSU) processome component, homolog (yeast)): Located at 12q21.2 (Chr 12; 75941042-75941042).** Required for 40S ribosome biogenesis. Involved in nucleolar processing of pre-18S ribosomal RNA and ribosome assembly (inferred function based on sequence similarity).^82^ The region also includes the testosterone- and estrogen-sensitive GLIPR1, GLIPR1L1 and GLIPR1L2 genes, which encode proteins involved in male germ cell maturation and sperm-oocyte binding.^117-119^ Proposed to modify PCOS risk through metabolic mechanisms.^76^

**17.** **TOX3 (TOX high mobility group box family member 3): Located at 16q12.1.** This gene regulates Ca2+-dependent neuronal transcription through interaction with the cAMP-response-element-binding protein (CREB).^120^ The protein encoded by this gene contains an HMG-box, indicating that it may be involved in bending and unwinding of DNA and alteration of chromatin structure (RefSeq). The C-terminus of the encoded protein is glutamine-rich due to CAG repeats in the coding sequence. A minor allele of this gene has been implicated in an elevated risk of breast cancer. In normal human tissues, TOX3 is largely expressed in the central nervous system (CNS), in the ileum, and within the brain in the frontal and occipital lobe. TOX3 overexpression induces transcription involving isolated estrogen-responsive elements and estrogen-responsive promoters, and protects neuronal cells from cell death caused by endoplasmic reticulum stress or BAX overexpression.^120^ *TOX3* has been highlighted as a potential PCOS susceptibility locus before and there is evidence it may modify the hyperandrogenemic aspects of the syndrome.^15,121^ Proposed to modify PCOS risk through inflammatory mechanisms.^76^

**18.** **MAPRE1 (Microtubule-associated protein, RP/EB family, member 1, also known as EB1): Located at 20q11.1-q11.23 (Chr 20; 31420757-31420757).** EB1 interacts with the low-density lipoprotein receptor related protein 1 (LRP1), which controls adipogenesis^28^ and may additionally mediate ovarian angiogenesis and follicle development.^29^ EB1 binds to the plus end of microtubules and regulates the dynamics of the microtubule cytoskeleton.^82^ It is thought that this protein is involved in suppression of microtubule dynamic instability, regulation of microtubule polymerization and spindle function, and chromosome stability (RefSeq).

**19. ARSD (arylsulfatase D): Located at Xp22.3 (X-chromosome; 2846021-2846021).** ARSD is a member of the sulfatase family and located within a cluster of similar arylsulfatase genes on chromosome X. The encoded proteins are essential for the correct composition of bone and cartilage matrix (RefSeq, GeneNetwork, USCS). This gene has been marked as a prognostic marker in chronic lymphocytic leukemia and has been suggested as a biological mechanism in chroinic lymphocytic leukemia – CLL.^122^ The Xp22.3 region also includes the gene for glycogenin 2 (GYG2), which is involved in glycogen biosynthesis and blood glucose homeostasis. It has been shown that glycogen biosynthesis pathways are impaired in PCOS.^123,124^

### **Supplementary note on gene enrichment analysis.**

We used MAGENTA (Meta-analysis Gene-set Enrichment of Variant Associations; version 2.4;^125^ and DEPICT (Data-driven Expression-Prioritized Integration for Complex Traits; release 142 for 1000 Genomes imputed data;^126^ ^127^ methods to specifically prioritize genes, pathways and tissues enriched in the genome-wide results of the PCOS meta-analysis. In brief, MAGENTA assesses the over-representation of genes with low P-values in their locus across manually curated databases. For the MAGENTA analysis, curated gene-sets and Gene Ontology (GO) gene-sets were obtained from the Molecular Signatures Database (MSigDB release v4.0;^128^). DEPICT prioritizes genes in the associated loci, detects enriched pathways and tissues based on derived data sets based on patterns of co-expression and the expression levels of the genes at associated loci. Further, we performed functional annotation enrichment analysis using GoShifter (Genomic Annotation Shifter;^129^). Functional annotations used in these analyses were transcription factor binding sites (172 transcription factors)^130^ and chromatin states in different tissues (n=196).^131^ We investigated whether the 14 PCOS-associated susceptibility variants detected in this study and the variants in LD with them (r^2^>0.6) co-localized with specific functional annotations. The results from gene set analysis did not show results that we found particularly trustworthy, and the methods of MAGENTA have been criticized elsewhere so these are only reported in the supplement. No individual pathway appeared to be significant. GoShifter analyses for identification of enriched functional annotations did not reveal any statistically significant finding (all p-value>0.05). DEPICT tissue identification approach reinforced the importance of ovarian morphology, with ovarian follicle, ovum, oocytes, ovary, granulosa cells, fallopian tubes and cumulus cells all showing nominally significant p-values (p-value<0.05). This is alongside the more general enrichment at endocrine cells and adipocytes.

The nominally significant findings for ovaries in the tissue identification analysis suggested the importance of ovarian morphology in PCOS pathogenesis. However, no individual pathway appeared to be significant in gene-set enrichment and gene prioritization analyses. A potential explanation for the lack of significant findings is that these methods are limited by the functional data available for the tissues relevant to PCOS and its related traits, e.g. ovary. In addition, DEPICT and GoShifter analyses were based on the 14 PCOS GWAS meta-analysis susceptibility variants, which may limit the power of these approaches to detect significant enrichments.^129^

**References**

71. Goodarzi, M.O. *et al.* Replication of association of DENND1A and THADA variants with polycystic ovary syndrome in European cohorts. *J Med Genet* **49**, 90-5 (2012).

72. Stuebe, A.M. *et al.* Obesity and diabetes genetic variants associated with gestational weight gain. *Am J Obstet Gynecol* **203**, 283 e1-17 (2010).

73. Cui, L. *et al.* Polycystic ovary syndrome susceptibility single nucleotide polymorphisms in women with a single PCOS clinical feature. *Hum Reprod* **30**, 732-6 (2015).

74. Brower, M.A. *et al.* Further investigation in europeans of susceptibility variants for polycystic ovary syndrome discovered in genome-wide association studies of Chinese individuals. *J Clin Endocrinol Metab* **100**, E182-6 (2015).

75. Cui, L. *et al.* Genotype-phenotype correlations of PCOS susceptibility SNPs identified by GWAS in a large cohort of Han Chinese women. *Hum Reprod* **28**, 538-44 (2013).

76. Pau, C.T., Mosbruger, T., Saxena, R. & Welt, C.K. Phenotype and Tissue Expression as a Function of Genetic Risk in Polycystic Ovary Syndrome. *PLoS One* **12**, e0168870 (2017).

77. Haskins, J.W., Nguyen, D.X. & Stern, D.F. Neuregulin 1-activated ERBB4 interacts with YAP to induce Hippo pathway target genes and promote cell migration. *Sci Signal* **7**, ra116 (2014).

78. Sudol, M. Neuregulin 1-activated ERBB4 as a "dedicated" receptor for the Hippo-YAP pathway. *Sci Signal* **7**, pe29 (2014).

79. Xiang, C. *et al.* Hippo signaling pathway reveals a spatio-temporal correlation with the size of primordial follicle pool in mice. *Cell Physiol Biochem* **35**, 957-68 (2015).

80. Sarikaya, D.P. & Extavour, C.G. The Hippo pathway regulates homeostatic growth of stem cell niche precursors in the Drosophila ovary. *PLoS Genet* **11**, e1004962 (2015).

81. Hsueh, A.J., Kawamura, K., Cheng, Y. & Fauser, B.C. Intraovarian control of early folliculogenesis. *Endocr Rev* **36**, 1-24 (2015).

82. The UniProt, C. UniProt: the universal protein knowledgebase. *Nucleic Acids Res* **45**, D158-D169 (2017).

83. Streets, A.J. *et al.* Parallel microarray profiling identifies ErbB4 as a determinant of cyst growth in ADPKD and a prognostic biomarker for disease progression. *Am J Physiol Renal Physiol* **312**, F577-F588 (2017).

84. Cohen, S. *et al.* Interferon regulatory factor 1 is an independent predictor of platinum resistance and survival in high-grade serous ovarian carcinoma. *Gynecol Oncol* **134**, 591-8 (2014).

85. Garcia-Reyero, N. *et al.* Expression signatures for a model androgen and antiandrogen in the fathead minnow (Pimephales promelas) ovary. *Environ Sci Technol* **43**, 2614-9 (2009).

86. Grenon, M., Gilbert, C. & Lowndes, N.F. Checkpoint activation in response to double-strand breaks requires the Mre11/Rad50/Xrs2 complex. *Nat Cell Biol* **3**, 844-7 (2001).

87. Zhang, Y., Zhou, J. & Lim, C.U. The role of NBS1 in DNA double strand break repair, telomere stability, and cell cycle checkpoint control. *Cell Res* **16**, 45-54 (2006).

88. Adelman, C.A., De, S. & Petrini, J.H. Rad50 is dispensable for the maintenance and viability of postmitotic tissues. *Mol Cell Biol* **29**, 483-92 (2009).

89. Bennett, J., Baumgarten, S.C. & Stocco, C. GATA4 and GATA6 silencing in ovarian granulosa cells affects levels of mRNAs involved in steroidogenesis, extracellular structure organization, IGF-I activity, and apoptosis. *Endocrinology* **154**, 4845-58 (2013).

90. Bennett, J., Wu, Y.G., Gossen, J., Zhou, P. & Stocco, C. Loss of GATA-6 and GATA-4 in granulosa cells blocks folliculogenesis, ovulation, and follicle stimulating hormone receptor expression leading to female infertility. *Endocrinology* **153**, 2474-85 (2012).

91. Padua, M.B., Fox, S.C., Jiang, T., Morse, D.A. & Tevosian, S.G. Simultaneous gene deletion of gata4 and gata6 leads to early disruption of follicular development and germ cell loss in the murine ovary. *Biol Reprod* **91**, 24 (2014).

92. Salilew-Wondim, D. *et al.* Polycystic ovarian syndrome is accompanied by repression of gene signatures associated with biosynthesis and metabolism of steroids, cholesterol and lipids. *J Ovarian Res* **8**, 24 (2015).

93. Lighvani, S. *et al.* Regulation of macrophage migration by a novel plasminogen receptor Plg-R KT. *Blood* **118**, 5622-30 (2011).

94. Burnicka-Turek, O. *et al.* Inactivation of insulin-like factor 6 disrupts the progression of spermatogenesis at late meiotic prophase. *Endocrinology* **150**, 4348-57 (2009).

95. Sumpter, R., Jr. *et al.* Fanconi Anemia Proteins Function in Mitophagy and Immunity. *Cell* **165**, 867-81 (2016).

96. Spicer, L.J. *et al.* The hedgehog-patched signaling pathway and function in the mammalian ovary: a novel role for hedgehog proteins in stimulating proliferation and steroidogenesis of theca cells. *Reproduction* **138**, 329-39 (2009).

97. McAllister, J.M. *et al.* Overexpression of a DENND1A isoform produces a polycystic ovary syndrome theca phenotype. *Proc Natl Acad Sci U S A* **111**, E1519-27 (2014).

98. McAllister, J.M., Legro, R.S., Modi, B.P. & Strauss, J.F., 3rd. Functional genomics of PCOS: from GWAS to molecular mechanisms. *Trends Endocrinol Metab* **26**, 118-24 (2015).

99. Laisk-Podar, T., Kaart, T., Peters, M. & Salumets, A. Genetic variants associated with female reproductive ageing--potential markers for assessing ovarian function and ovarian stimulation outcome. *Reprod Biomed Online* **31**, 199-209 (2015).

100. Ruth, K.S. *et al.* Genome-wide association study with 1000 genomes imputation identifies signals for nine sex hormone-related phenotypes. *Eur J Hum Genet* **24**, 284-90 (2016).

101. Grigorova, M. *et al.* Reproductive physiology in young men is cumulatively affected by FSH-action modulating genetic variants: FSHR -29G/A and c.2039 A/G, FSHB -211G/T. *PLoS One* **9**, e94244 (2014).

102. Hagen, C.P. *et al.* FSHB-211 and FSHR 2039 are associated with serum levels of follicle-stimulating hormone and antimullerian hormone in healthy girls: a longitudinal cohort study. *Fertil Steril* **100**, 1089-95 (2013).

103. Schuring, A.N., Busch, A.S., Bogdanova, N., Gromoll, J. & Tuttelmann, F. Effects of the FSH-beta-subunit promoter polymorphism -211G->T on the hypothalamic-pituitary-ovarian axis in normally cycling women indicate a gender-specific regulation of gonadotropin secretion. *J Clin Endocrinol Metab* **98**, E82-6 (2013).

104. Tong, Y., Liao, W.X., Roy, A.C. & Ng, S.C. Association of AccI polymorphism in the follicle-stimulating hormone beta gene with polycystic ovary syndrome. *Fertil Steril* **74**, 1233-6 (2000).

105. Lee, H. *et al.* Genome-wide association study identified new susceptibility loci for polycystic ovary syndrome. *Hum Reprod* **30**, 723-31 (2015).

106. Matthews, C.H. *et al.* Primary amenorrhoea and infertility due to a mutation in the beta-subunit of follicle-stimulating hormone. *Nat Genet* **5**, 83-6 (1993).

107. Kumar, T.R., Wang, Y., Lu, N. & Matzuk, M.M. Follicle stimulating hormone is required for ovarian follicle maturation but not male fertility. *Nat Genet* **15**, 201-4 (1997).

108. Li, T. *et al.* Identification of YAP1 as a novel susceptibility gene for polycystic ovary syndrome. *J Med Genet* **49**, 254-7 (2012).

109. Louwers, Y.V., Stolk, L., Uitterlinden, A.G. & Laven, J.S. Cross-ethnic meta-analysis of genetic variants for polycystic ovary syndrome. *J Clin Endocrinol Metab* **98**, E2006-12 (2013).

110. Mariani, F. *et al.* PLZF expression during colorectal cancer development and in normal colorectal mucosa according to body size, as marker of colorectal cancer risk. *ScientificWorldJournal* **2013**, 630869 (2013).

111. Todd, J.A. *et al.* Robust associations of four new chromosome regions from genome-wide analyses of type 1 diabetes. *Nat Genet* **39**, 857-64 (2007).

112. Mukherjee, A. & Roy, S.K. Expression of ErbB3-binding protein-1 (EBP1) during primordial follicle formation: role of estradiol-17ss. *PLoS One* **8**, e67068 (2013).

113. Saxena, R. *et al.* Han Chinese polycystic ovary syndrome risk variants in women of European ancestry: relationship to FSH levels and glucose tolerance. *Hum Reprod* **30**, 1454-9 (2015).

114. Yun, H.J. *et al.* An early endosome regulator, Rab5b, is an LRRK2 kinase substrate. *J Biochem* **157**, 485-95 (2015).

115. Chen, P.I., Kong, C., Su, X. & Stahl, P.D. Rab5 isoforms differentially regulate the trafficking and degradation of epidermal growth factor receptors. *J Biol Chem* **284**, 30328-38 (2009).

116. Jones, M.R. *et al.* Systems genetics reveals the functional context of PCOS loci and identifies genetic and molecular mechanisms of disease heterogeneity. *PLoS Genet* **11**, e1005455 (2015).

117. Zhang, L.J. *et al.* Testosterone regulates thyroid cancer progression by modifying tumor suppressor genes and tumor immunity. *Carcinogenesis* **36**, 420-8 (2015).

118. Rasmussen, L.M. *et al.* Prolactin and oestrogen synergistically regulate gene expression and proliferation of breast cancer cells. *Endocr Relat Cancer* **17**, 809-22 (2010).

119. Gibbs, G.M. *et al.* Glioma pathogenesis-related 1-like 1 is testis enriched, dynamically modified, and redistributed during male germ cell maturation and has a potential role in sperm-oocyte binding. *Endocrinology* **151**, 2331-42 (2010).

120. Zhang, X. *et al.* A genetic polymorphism in TOX3 is associated with survival of gastric cancer in a Chinese population. *PLoS One* **8**, e72186 (2013).

121. Cui, Y. *et al.* Mutational analysis of TOX3 in Chinese Han women with polycystic ovary syndrome. *Reprod Biomed Online* **29**, 752-5 (2014).

122. Trojani, A. *et al.* Gene expression profiling identifies ARSD as a new marker of disease progression and the sphingolipid metabolism as a potential novel metabolism in chronic lymphocytic leukemia. *Cancer Biomark* **11**, 15-28 (2011).

123. Book, C.B. & Dunaif, A. Selective insulin resistance in the polycystic ovary syndrome. *J Clin Endocrinol Metab* **84**, 3110-6 (1999).

124. Wu, X.K. *et al.* Selective ovary resistance to insulin signaling in women with polycystic ovary syndrome. *Fertil Steril* **80**, 954-65 (2003).

125. Segre, A.V. *et al.* Common inherited variation in mitochondrial genes is not enriched for associations with type 2 diabetes or related glycemic traits. *PLoS Genet* **6**(2010).

126. Pers, T.H. *et al.* Biological interpretation of genome-wide association studies using predicted gene functions. *Nat Commun* **6**, 5890 (2015).

127. Genomes Project, C. *et al.* A map of human genome variation from population-scale sequencing. *Nature* **467**, 1061-73 (2010).

128. Subramanian, A. *et al.* Gene set enrichment analysis: a knowledge-based approach for interpreting genome-wide expression profiles. *Proc Natl Acad Sci U S A* **102**, 15545-50 (2005).

129. Trynka, G. *et al.* Disentangling the Effects of Colocalizing Genomic Annotations to Functionally Prioritize Non-coding Variants within Complex-Trait Loci. *Am J Hum Genet* **97**, 139-52 (2015).

130. Consortium, E.P. An integrated encyclopedia of DNA elements in the human genome. *Nature* **489**, 57-74 (2012).

131. Roadmap Epigenomics Consortium *et al.* Integrative analysis of 111 reference human epigenomes. *Nature* **518**, 317-30 (2015).
