# Supplementary material for: Large-scale genome-wide meta-analysis of polycystic ovary syndrome suggests shared genetic architecture for different diagnosis criteria
Source: PLoS Genet. 2018 Dec 19;14(12):e1007813. doi: 10.1371/journal.pgen.1007813 (PMC6300389; doi:10.1371/journal.pgen.1007813)
Supplement: S2 Fig — (DOCX) [file pgen.1007813.s010.docx]

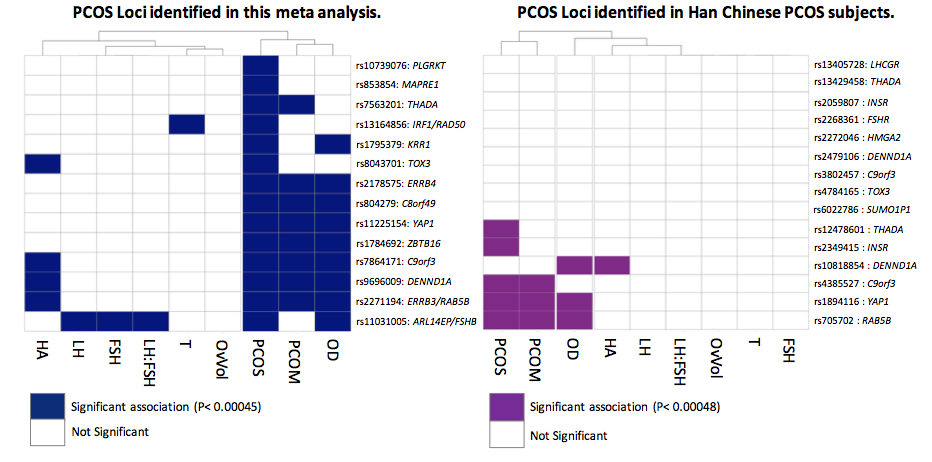


**Supplementary Figure 2. Cluster plots showing relationships between PCOS loci and related traits.** Loci significantly associated with PCOS in our meta-analysis (shown on left, blue) or in the previously reported meta analyses of Chinese PCOS subjects in the analysis of related traits in our own meta-analysis (shown on right, purple). Clustering by column (phenotype/trait) demonstrates the large proportion of PCOS loci that are also significantly associated with ovulatory dysfunction (OD) and polycystic ovarian morphology (PCOM).
