# Supplementary material for: Large-scale genome-wide meta-analysis of polycystic ovary syndrome suggests shared genetic architecture for different diagnosis criteria
Source: PLoS Genet. 2018 Dec 19;14(12):e1007813. doi: 10.1371/journal.pgen.1007813 (PMC6300389; doi:10.1371/journal.pgen.1007813)
Supplement: S3 Fig — (DOCX) [file pgen.1007813.s011.docx]

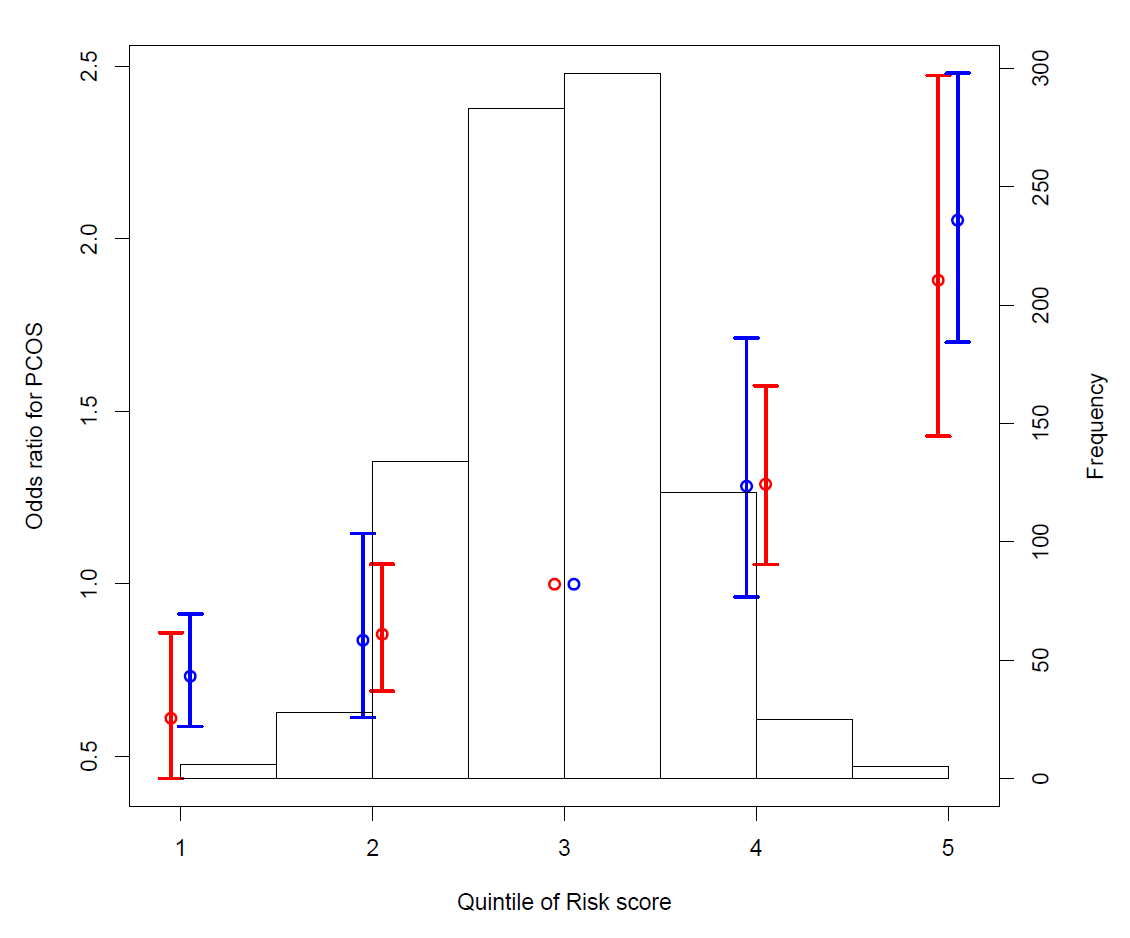
**Supplementary Figure 3: Weighted genetic risk score.** The odds for having PCOS by NIH (red) or Rotterdam (blue) diagnostic criteria based on genetic risk score from across the identified genome-wide significant loci. The group with the average number of risk alleles was used as the reference group.
