# Supplementary figures and images for: Large-scale genome-wide meta-analysis of polycystic ovary syndrome suggests shared genetic architecture for different diagnosis criteria
Source: PLoS Genet. 2018 Dec 19;14(12):e1007813. doi: 10.1371/journal.pgen.1007813 (PMC6300389; doi:10.1371/journal.pgen.1007813)

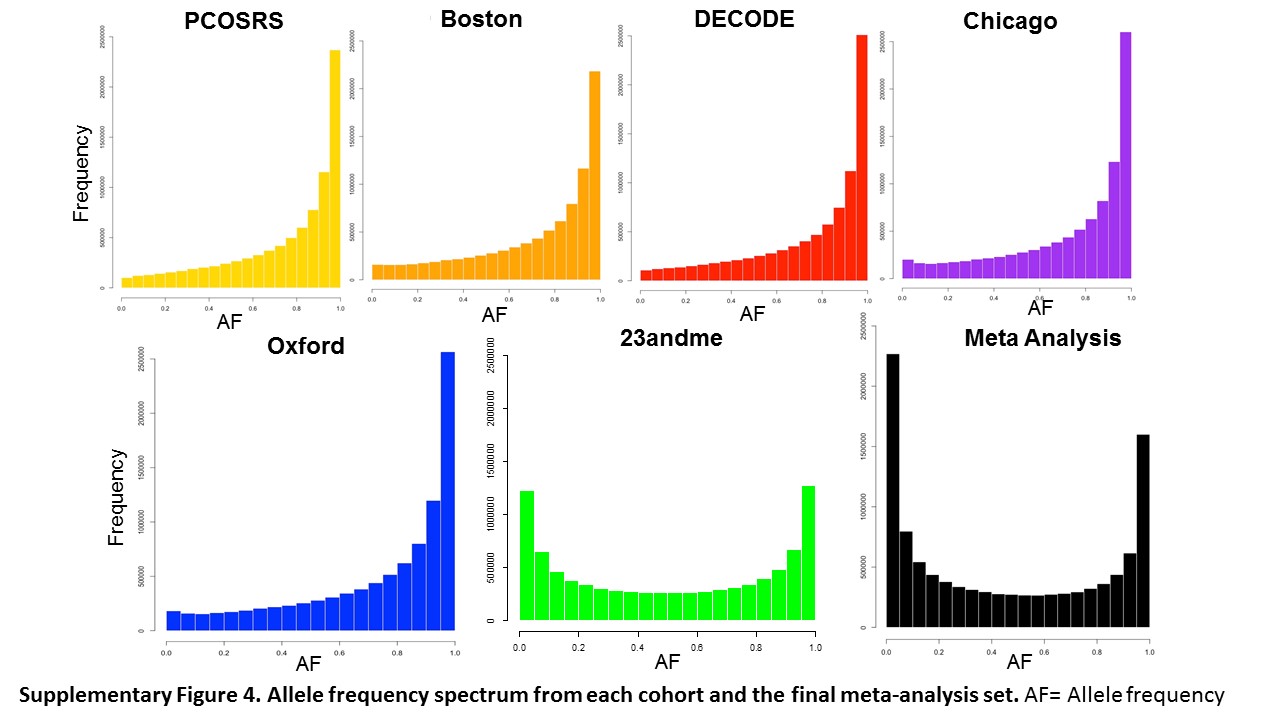

Supplement: S4 Fig — (DOCX) [file pgen.1007813.s012.docx]
